# Wild and Captive Environments Drive the Convergence of Gut Microbiota and Impact Health in Threatened Equids

**DOI:** 10.3389/fmicb.2022.832410

**Published:** 2022-06-23

**Authors:** Zhichao Zhou, Liping Tang, Liping Yan, Huiping Jia, Yu Xiong, Jin Shang, Changliang Shao, Qiangwei Zhang, Hongjun Wang, Lun He, Defu Hu, Dong Zhang

**Affiliations:** ^1^School of Ecology and Nature Conservation, Beijing Forestry University, Beijing, China; ^2^Mt. Kalamaili Ungulate Nature Reserve, Changji, China; ^3^Gansu Endangered Animals Protection Center, Wuwei, China; ^4^China Wildlife Conservation Association, Beijing, China

**Keywords:** gut microbiota, sympatry, microbiome convergence, wild and captive, equid, health

## Abstract

To explore how the living environment influences the establishment of gut microbiota in different species, as well as the extent to which changes in the living environment caused by captive breeding affect wildlife’s gut microbiota and health, we used 16S rRNA gene amplicon sequencing and shotgun metagenomic sequencing to compare the gut microbiome of two species of threatened equids, the Przewalski’s Horse and the Asian wild ass, in the wild and captivity. The results revealed that different species of Equidae living in the same environment showed remarkable convergence of gut microflora. At the same time, captive populations exhibited significantly “unhealthy” microbiota, such as low Alpha diversity, high levels of potentially pathogenic bacteria and biomarkers of physical or psychological disease, and enrichment of microbial functions associated with exogenous exposure and susceptibility, implying that the artificial environment created by captivity may adversely impact the health of wildlife to some extent. Our findings demonstrate the importance of the environmental factors for the establishment of gut microbiota and host health and provide new insights into the conservation of wildlife in captivity from the perspective of the microbiome.

## Introduction

The mammalian gut is home to a large number of symbiotic microorganisms that play a vital role in maintaining the normal life processes of the host animal, such as nutrient digestion and absorption, pathogen prevention, and toxic compound degradation (Roggenbuck et al., 2014; Hanning and Diaz-Sanchez, 2015; Blyton et al., 2019; Li et al., 2021; Peixoto et al., 2021), and even the ability of gut microbiota to produce neuroactive molecules that affect the host animal’s brain, thus causing alterations in mood and behavior (Vuong et al., 2017; Sarkar et al., 2018). The establishment of commensal microbiota is usually affected by the phylogeny of the host which means that taxonomically different hosts tend to form their unique microflora structure during the long-term evolutionary process (Ley et al., 2008; Groussin et al., 2017). However, there has been accumulating evidence in recent years that environmental factors such as diet, habitat, and lifestyle play a dominant role in the composition of host gut microbiota (Zarrinpar et al., 2014; Ren et al., 2017; Kartzinel et al., 2019). Changes in the living environment can have a significant impact on the host’s gut microbiota. Jin et al. (2021) demonstrated that as carnivores, giant pandas emerged with numerous lignin-digesting microbial taxa in their gut microbiota to adapt to bamboo as a special diet. Some studies even suggest that the same living environment might drive the gut microflora of distinct host species to convergence (Muegge et al., 2011; Angelakis et al., 2016; Clayton et al., 2016; Greene et al., 2019). For instance, a study on herbivores showed that the gut microbiota of yaks and Tibetan sheep, which share a high altitude, converged to adapt to the harsh plateau environment (Zhang et al., 2016). In non-human primates, sympatric gorillas and chimpanzees also have similar gut microflora (Moeller et al., 2013).

As an essential way to conserve threatened wildlife, captive breeding can effectively prevent species extinction (Bowkett, 2009). At the same time, the dramatic change in the living environment undergone by wildlife as they transfer from the wild to captivity becomes a quintessential model for exploring how environmental factors affect the gut microbiota of wildlife (McKenzie et al., 2017). Furthermore, numerous studies have confirmed that host microbiota disorders due to environmental alterations may cause various metabolic diseases in hosts, such as obesity, irritable bowel syndrome (IBS), and inflammatory bowel disease (IBD; Cox et al., 2015; Shreiner et al., 2015). Therefore, we can further understand the effects of captive breeding on wildlife health by examining differences in gut microbiota composition caused to environmental changes (West et al., 2019; Zhu et al., 2021). However, current comparative microbiological studies of wildlife in the wild and captivity have focused on single species (Gibson et al., 2019; Schmidt et al., 2019; Sun et al., 2019). Rarely have multiple species been compared simultaneously to examine whether uniform conditions of captivity have similar impacts on the gut microbiota of different species.

Therefore, we utilized 16S rRNA gene amplicon sequencing and shotgun metagenomic sequencing in this study to compare the diversity, composition, and function of the gut microbiome of two threatened Equidae species, the Przewalski’s Horse (*Equus ferus przewalskii*; PH) and the Asian wild ass (*Equus hemionus*; AWA), living in the wild and captive environments. To explore the effects of field sympatry and uniform captive environments on the gut microbiota of different species of Equidae and reveal the potential impact of anthropogenic alterations in the living environment on the health of threatened equids from the perspective of the microbiome, allowing for a more comprehensive understanding of the captive breeding program for wildlife conservation management.

## Materials and Methods

### Study Area and Sample Collection

All fecal samples were collected from the adult in September of the same year to avoid the effect of ages and different seasons on the microbiota of equids (Supplementary Table 1). Fresh fecal samples of PHs (*n* = 12) and AWAs (*n* = 10) in the wild environment were collected on 21–22 September 2020 at Kalamaili Nature Reserve (KNR), located in the Altay Prefecture of Xinjiang Uygur Autonomous Region, China (44°36′-46°00′ N, 88°30′-90°03′ E). Fresh fecal samples of PHs (*n* = 13) and AWAs (*n* = 6) in the captive environment were collected on 26 September 2020 at Gansu Endangered Animals Protection Center (GEAPC) in Wuwei, Gansu Province, China (37°52′50″ N, 102°52′48″ E). The KNR is geographically located in the arid desert region of the Junggar Basin, covering an area of 18,000 km^2^, where PHs and AWAs roam freely, mainly feeding on bunchgrasses like needlegrass (*Stipa capillata*) and subshrubs such as Pamirian winterfat (*Krascheninnikovia ceratoides*), wormwood (*Artemisia* spp.), and *Anabasis brevifolia* that distributed in reserve (Meng, 2007). The GEAPC lies 1,400 km from KNR, at the southern edge of the Tengger Desert, where PHs and AWAs are kept in enclosures and fed daily with alfalfa (*Medicago sativa*) hay. Fresh feces samples were collected into sterile centrifuge tubes, labeled, and immediately placed in a portable fridge before being transported to the laboratory and stored at −20°C until DNA extraction.

The animal study was reviewed and approved by the Ethics and Animal Welfare Committee of Beijing Forestry University (EAWC_BJFU_2021012). Fecal sample collection was approved by the WHBRC and GEAPC.

### DNA Extraction

Total DNA extraction from the microbial community of feces samples was performed according to the manufacturer’s instructions of the E.Z.N.A.® soil DNA kit (Omega Bio-tek, Norcross, GA, United States). The TissueLyser (Onebio tech, China) was used to add a mechanical disruption stage of bacterial cells by bead-beating using disruptor tubes (Omega Bio-tech, United States) at a frequency of 45 Hz for 250 s. The quality of extracted DNA was examined using 1% agarose gel electrophoresis, and DNA concentration and purity were determined with NanoDrop 2000.

### 16S rRNA Gene Amplicon Sequencing and Data Processing

PCR amplification of the V3-V4 hyper-variable region of the 16S rRNA gene of all DNA samples was carried out using 338F and 806R (Mori et al., 2014) with the following amplification procedure: Initial denaturation at 95°C for 3 min, 27 cycles (denaturation at 95°C for 30 s, annealing at 55°C for 30 s, elongation at 72°C for 45 s), followed by extension at 72°C for 10 min, and finally storage at 4°C. The PCR mixtures consist of template DNA 10 ng, 0.8 μl of 5 μM of each forward and reverse primer, 2 μl of 2.5 mM dNTPs, 0.4 μl of *TransStart* FastPfu DNA polymerase, 4 μl of 5 × *TransStart* FastPfu buffer, and finally add ddH_2_O supplement to 20 μl. PCR reaction of each sample was performed in triplicate. The PCR products of each sample were mixed and extracted from 2% agarose gel, then purified using the AxyPrep DNA Gel Extraction Kit (Axygen Biosciences, Union City, CA, United States) according to manufacturer’s instructions, detected by 2% agarose gel electrophoresis, and quantified using the QuantiFluor® dsDNA System (Promega, United States). Purified amplicons were pooled in equimolar and paired-end sequenced on an Illumina Miseq PE300 platform. 16S rRNA gene amplicon sequencing data were deposited into the NCBI Sequence Read Archive (SRA) database under accession numbers PRJNA784453 (AWAs and captive PHs) and PRJNA701711 (wild PHs).

The raw 16S rRNA gene sequencing reads were quality-filtered by fastp (version 0.20.0; Chen et al., 2018) and merged by FLASH (version 1.2.11; Magoč and Salzberg, 2011) with a minimum overlap length of 10 bp. Operational taxonomic units (OTUs) were clustered with a 97% similarity criterion using UPARSE (version 7.1; Edgar, 2013), and chimeric sequences were identified and eliminated. The taxonomy of each OTU representative sequence was annotated by RDP Classifier (version 2.11; Wang et al., 2007) against the Silva 16S rRNA database (v138) with a confidence threshold of 70%.

### Shotgun Metagenomic Sequencing and Bioinformatics Analysis

Shotgun metagenomic sequencing was carried out on the same DNA extracts as the 16S rRNA analysis. Twelve samples (three each for wild PHs, wild AWAs, captive PHs, and captive AWAs) with high DNA quality were selected. Metagenomic DNA was broken into about 400 bp fragments with Covaris M220 (Gene Company Limited, China). A paired-end library was then constructed using NEXTFLEX Rapid DNA-Seq (Bioo Scientific, Austin, TX, United States). Paired-end sequencing was performed using the NovaSeq Reagent Kits on the NovaSeq 6,000 platform (Illumina Inc., San Diego, CA, United States). Metagenomic sequence data for this project have been deposited in the NCBI Sequence Read Archive (Accession Number: PRJNA784453).

The raw metagenomic data were quality-filtered using the Fastp (version 0.20.0), whereby the adapters were eliminated and reads with low quality (*Q* < 20) and shorter than 20 bp were removed. Reads that mapped to the PH and AWA genome were also filtered out using BWA (version 0.7.17; Li and Durbin, 2009). Metagenomic data were assembled using MEGAHIT (version 1.1.2; Li et al., 2015) with the minimum contig length set to 300 bp. A non-redundant gene catalog was constructed with CD-HIT (version 4.6.1; Fu et al., 2012) with a 90% sequence identity (90% coverage). Bacterial and archaeal genes were filtered from the non-redundant gene catalog to create a prokaryotic gene catalog. High-quality reads were aligned against the prokaryotic gene catalog with 95% identity using SOAPaligner (version 2.21; Li et al., 2009) to determine the gene abundance in each sample. Representative sequences were mapped to the Kyoto Encyclopedia of Genes and Genomes (KEGG) database using Diamond (version 0.8.35; Buchfink et al., 2014) for functional annotations with an e-value of 1e-5.

### Statistical Analysis

16S rRNA gene amplicon sequences were rarefied at a minimum sequencing depth before further analysis and obtained the practical OTU set. Alpha diversity of each sample was calculated using mothur (version 1.30.2; Schloss et al., 2009), and R presented Shannon rarefaction curves. The Wilcoxon rank-sum test was utilized to detect a statistically significant difference in Alpha diversity between groups. R was used to count the bacterial abundance of each sample at each taxonomic level and to visualize the bacterial community composition. The QIIME pipeline (Caporaso et al., 2010) was applied to calculate the beta diversity metrics, and NMDS analysis was performed using the R *Vegan* package. The differences between groups were tested via ANOSIM analysis with a Monte Carlo permutation test (999 permutations). Linear discriminant analysis (LDA) effect size (LEfSe) analyses were performed using LEfSe software^1^ (Segata et al., 2011) to compare the microbial composition differences between wild and captive equids and to compare the metagenomic functional differences based on the Kyoto Encyclopedia of Genes and Genomes (KEGG) database.

## Results

### Sequence Statistics

A total of 2,064,221 raw reads were obtained after 16S rRNA gene sequencing of 41 fecal samples from twelve wild PHs, ten captive PHs, thirteen wild AWAs, and six captive AWAs, with an average of 50,347 ± 8,304 reads per sample, and there were 1,007,730 reads (24,580 reads per sample) after quality control and sequences rarefied. A total of 3,660 OTUs were obtained after a 97% clustering threshold and classified into 25 phyla, 57 classes, 142 orders, 258 families, and 561 genera of bacteria. Shotgun metagenomic sequencing produced a total of 892,958,226 reads from 12 samples, with an average of 74,413,186 ± 4,493,192 reads per sample. In total, 5,093,229 catalog genes were constructed with an average sequence length of 539 bp in the non-redundant gene catalog.

### Microbial Diversity Analysis

**Figure 1:**
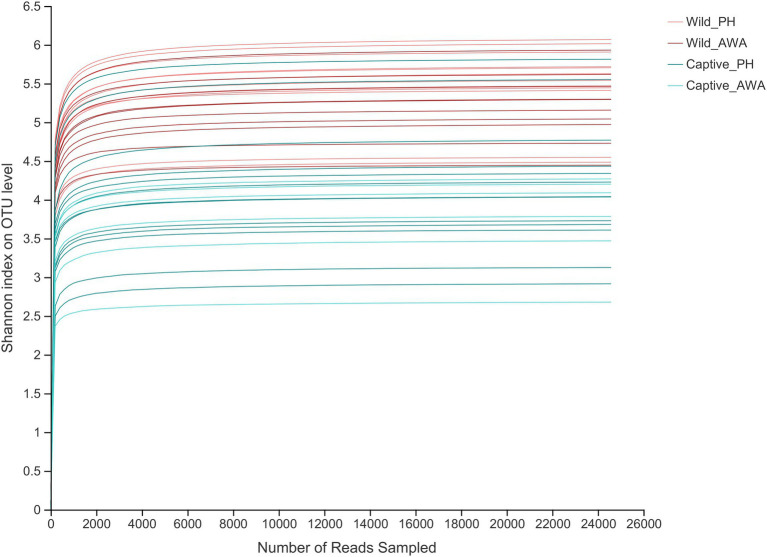
Shannon index’s Alpha diversity rarefaction curves on the OTU level. The abscissa represents the number of reads selected randomly; the ordinate represents the Alpha diversity measured.

**Figure 2:**
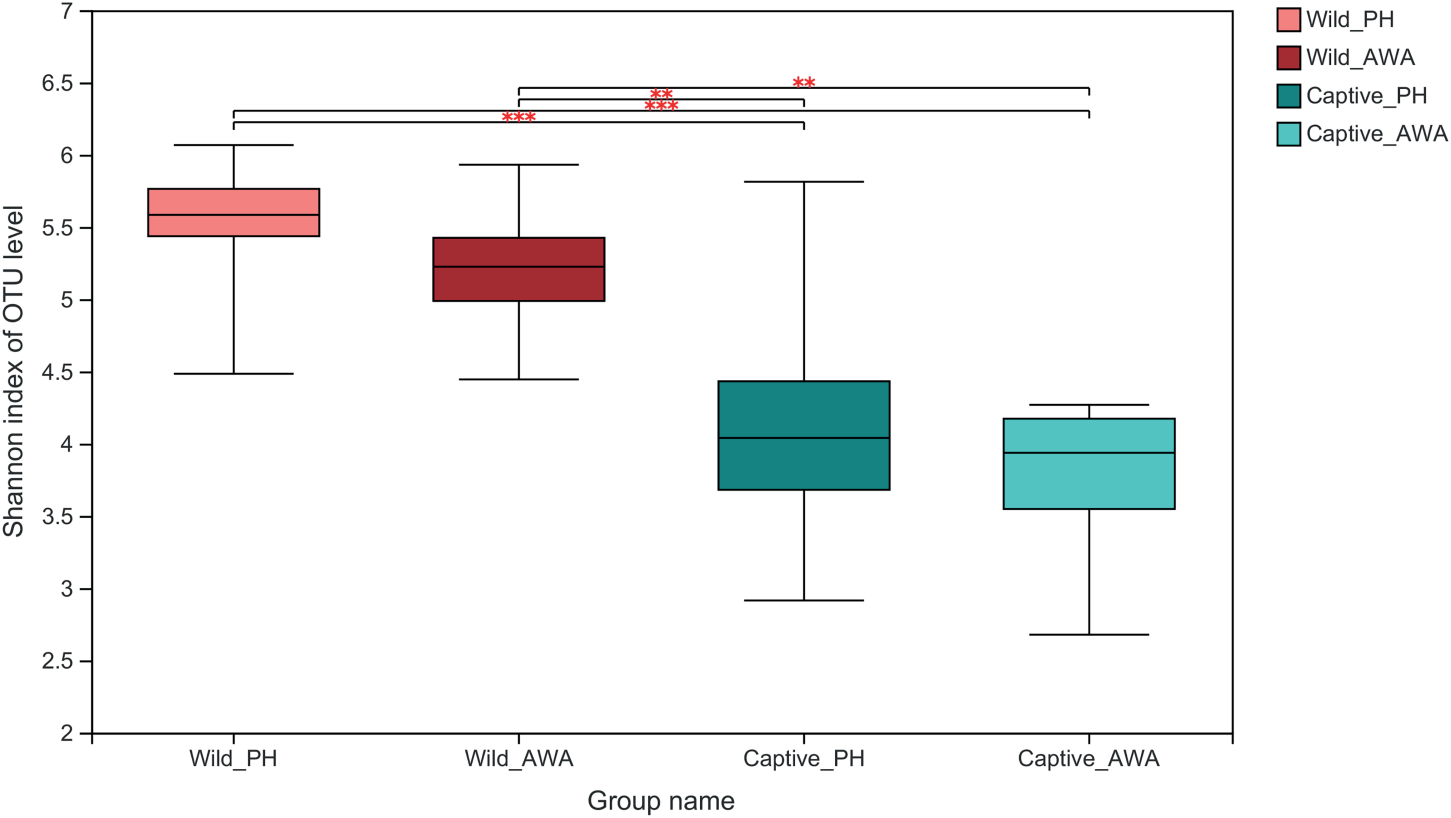
Wilcoxon rank-sum test for differences in Alpha diversity (Shannon index) between groups on the OTU level. ^**^p < 0.01 and ^***^p < 0.001.

There were varying levels of Alpha diversity exhibited across PHs and AWAs in different living environments. The Shannon rarefaction curves revealed that each sample’s Shannon diversity increased and eventually leveled off with increasing sequencing depth, indicating the amount of sequencing data is adequate; nevertheless, the Shannon diversity of captive PHs and captive AWAs presented lower levels (Figure 1). The Wilcoxon rank-sum test was utilized to determine the richness (Chao index), diversity (Shannon index), and evenness (Shannon even index) of the gut microbial communities of wild and captive equids. There were significant differences in the Alpha diversity of equid gut microbiota between the two living environments. Except for microbial richness between AWAs, practically all diversity indexes exhibited that the wild population was significantly higher than the captive population. However, there was no significant difference in the microflora Alpha diversity between the two species of Equidae under the same living environment (i.e., wild or captive; Figure 2). These results indicate that the same survival environment confers convergent gut microbial Alpha diversity to PHs and AWAs, although the wild environment manifests a higher contribution in maintaining high Alpha diversity of gut microbiota.

**Figure 3:**
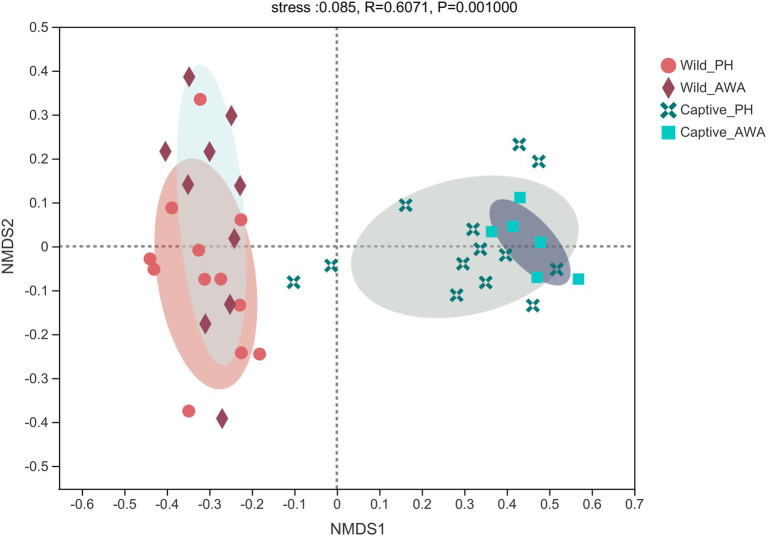
Non-metric multidimensional scaling (NMDS) plot of the gut microbiota in equids on the OTU level. The closer the points, the more similar of microbial community composition.

The Beta diversity of the two species of Equidae was calculated at the OTU level by the NMDS analysis based on weighted UniFrac distance (stress: 0.085). The results displayed that the microbiota composition of cohabiting PHs and AWAs was clustered, whereas the microflora structure of equids living in different environments had apparent separation (Figure 3).

### Microbial Composition Analysis

**Figure 4:**
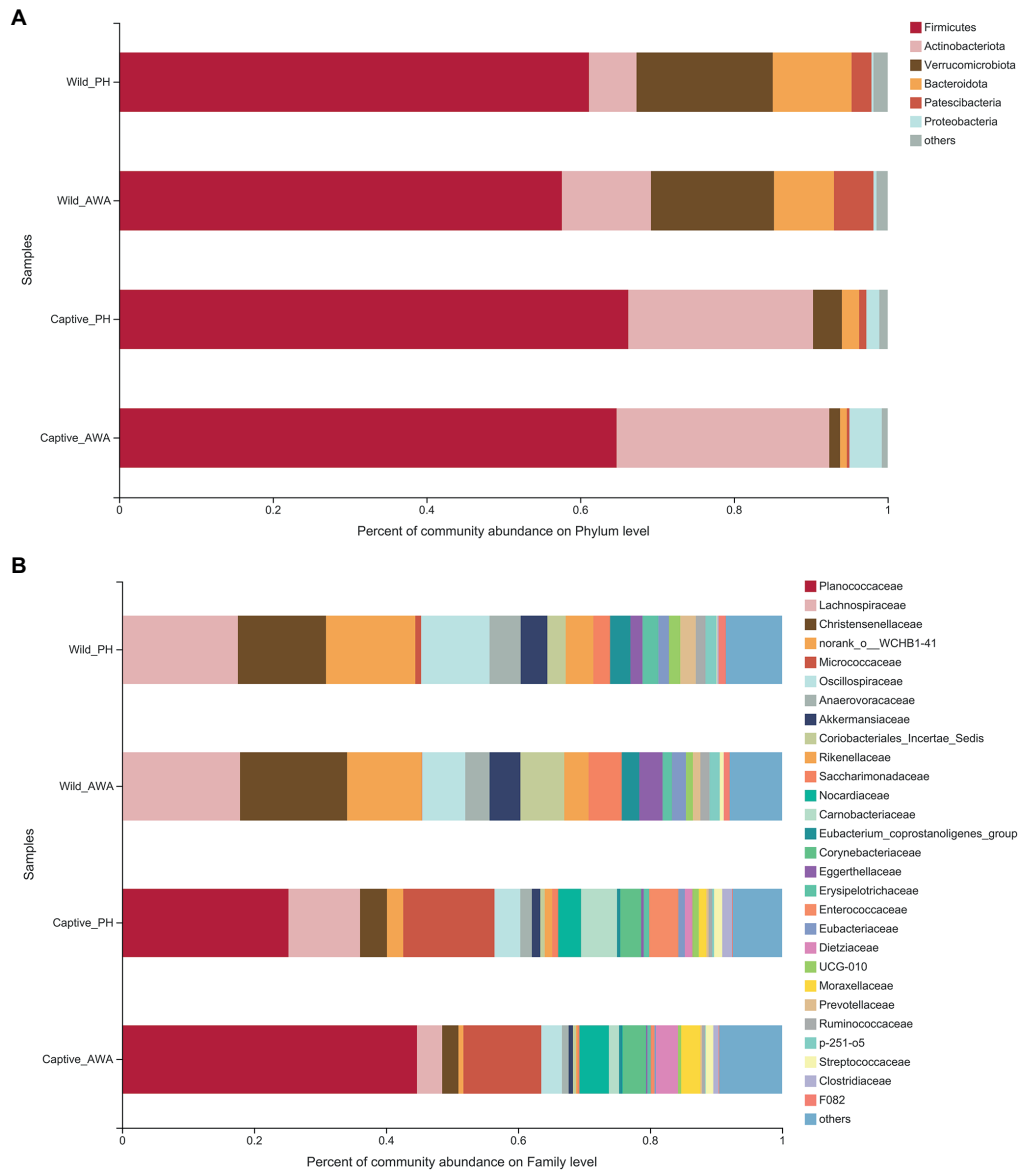
Fecal microbial community composition on the Phylum **(A)** and family **(B)** level in equids in the wild and captivity and values are averaged within groups.

The analysis of microbial community composition demonstrated that PHs and AWAs inhabiting the same environment had convergent microbiota structures, whereas the microflora of equids in different environments had significant differences. At the Phylum level, the gut microbiota of PHs and AWAs in the wild was primarily composed of *Firmicutes* (PH: 61.14%; AWA: 57.62%), *Verrucomicrobiota* (PH: 17.73%; AWA: 16.01%), and *Bacteroidota* (PH: 10.26%; AWA: 7.80%), while the microflora of equids in the captivity was principally constituted of *Firmicutes* (PH: 66.26%; AWA: 64.74%) and *Actinobacteriota* (PH: 24.03%; AWA: 27.66%). Meanwhile, equids in the captive environment had a higher ratio of *Firmicutes* to *Bacteroidota* (F/B; Figure 4A). At the Family level, the microbial composition of equids in the wild exhibited higher community evenness, with *Lachnospiraceae*, *Christensenellaceae*, *norank_o__WCHB1-41*, *Oscillospiraceae*, and *Akkermansiaceae* all accounting for considerable percentages. The symbiotic microbiota of equids in captivity, on the other hand, presented a fair preponderance of *Planococcaceae* and *Micrococcaceae* (Figure 4B).

**Figure 5:**
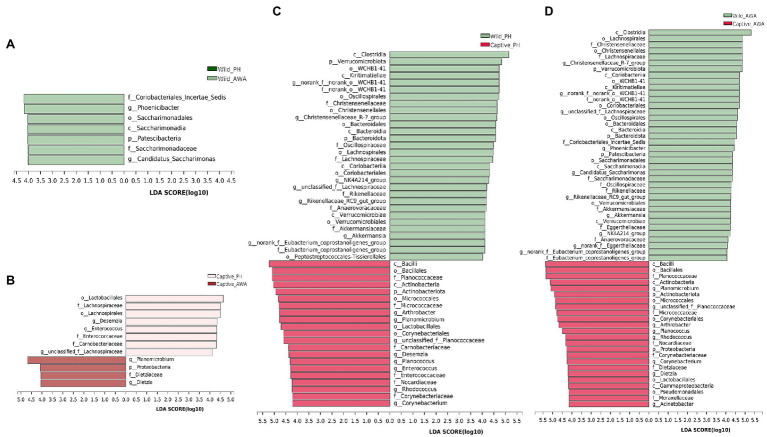
Linear discriminant analysis (LDA) effect size (LEfSe) analyses (LDA score threshold >4.0) on fecal microbial communities between wild PHs and AWAs **(A)**, captive PHs and AWAs **(B)**, wild and captive PHs **(C)**, and wild and captive AWAs **(D)**.

Linear discriminant analysis (LDA) effect size (LEfSe) analyses with the same LDA score threshold were performed on the microbial communities of PHs and AWAs in the wild and captivity, respectively, to explore the effects of environmental change on the relative abundance of gut microbiota in equids (Figure 5). The results exhibited that in comparison with equids living in different environments, different species of Equidae living in the same environment have fairly similar gut microbiota structures. In detail, only 7 (1 phylum, 1 class, 1 order, 2 families, 2 genera) and 12 taxa (1 phylum, 0 class, 2 orders, 4 families, 5 genera) displayed significant differences in relative abundance in the same living environment (wild or captive, respectively) of the gut microbiota between PHs and AWAs. However, the relative abundance of 51 (3 phyla, 7 classes, 12 orders, 14 families, 15 genera) and 62 taxa (5 phyla, 9 classes, 13 orders, 17 families, 18 genera) of the gut microbiota of PHs and AWAs, respectively, exhibited significant differences between different environments. Specifically, at the family level, *Lachnospiraceae*, *Christensenellaceae*, *norank_o__WCHB1-41*, *Oscillospiraceae*, *Akkermansiaceae*, *Rikenellaceae*, *Eubacterium_coprostanoligenes_group*, and Anaerovoracaceae exhibited higher relative abundance in wild PHs and AWAs. Meanwhile, Planococcaceae, Micrococcaceae, Nocardiaceae, and Corynebacteriaceae showed higher relative abundance in captive populations. On the side, *Carnobacteriaceae* and *Enterococcaceae* were abundant in captive PHs, whereas *Dietziaceae* and *Moraxellaceae* had a significantly higher trend in captive AWAs. These results suggested that the effect of environmental variation from wild to captive on the composition and abundance of microflora in PHs and AWAs were remarkably comparable. In other words, cohabitation converged the establishment of the gut microbiota in different Equidae species.

### Microbial Functional Analysis

**Figure 6:**
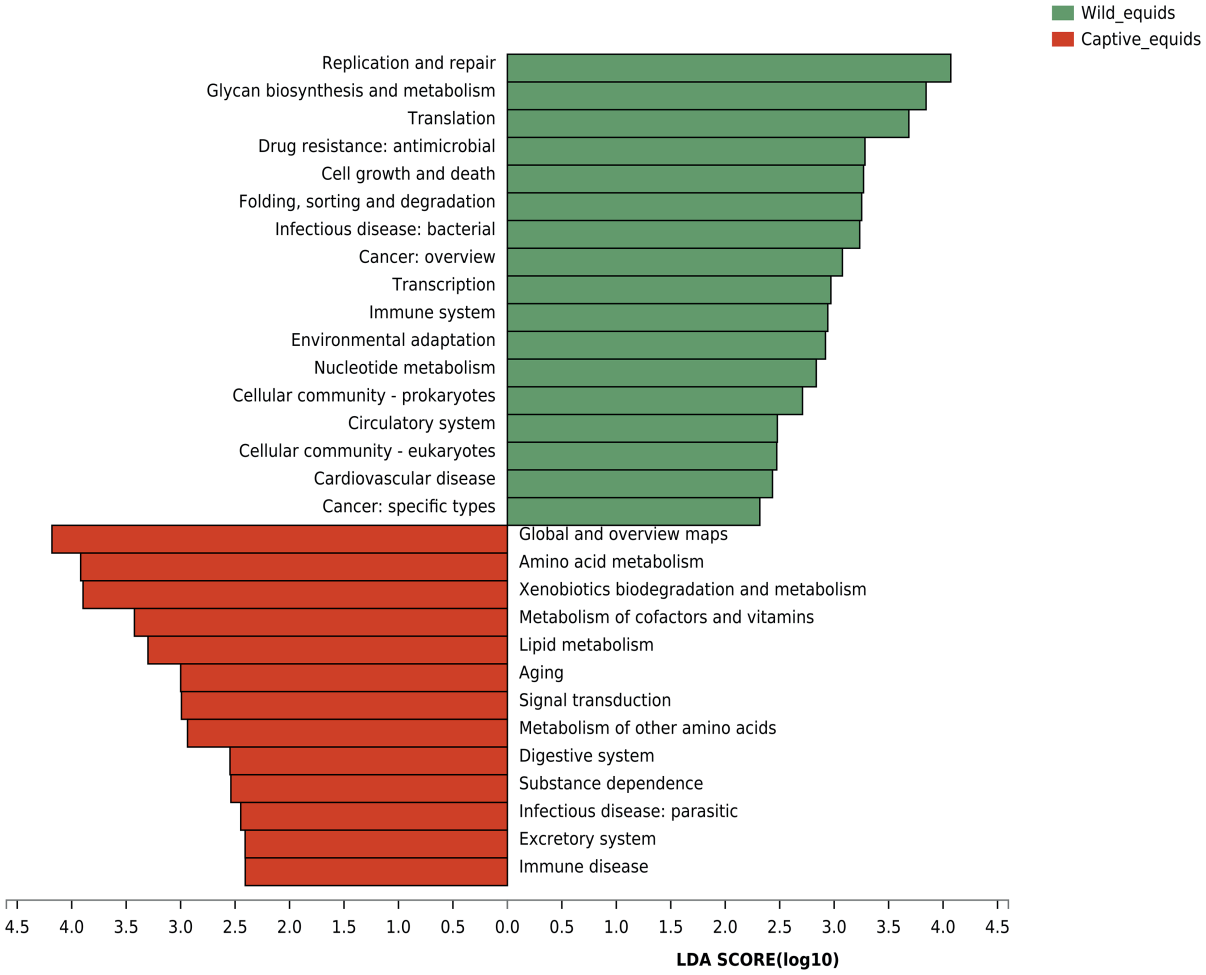
Linear discriminant analysis (LDA) effect size (LEfSe) analyses (LDA score threshold >2.0) of KEGG functional pathways for the microbial metagenome of the wild and captive equids.

Although PHs and AWAs living in the same environment have remarkably similar gut microbiota structures, equids in different environments have significantly different microflora. Therefore, based on the Kyoto Encyclopedia of Genes and Genomes (KEGG) database, the gut microbiome function (KEGG pathway level 2) of equids living in the wild and captivity was analyzed by the linear discriminant analysis (LDA) effect size (LEfSe) algorithm to investigate how this variation in microbiota structure would affect the functional pathway (Figure 6). The results indicated that the abundance of pathways for digestion and utilization of nutrients, including “Digestive system,” “Amino acid metabolism,” “Metabolism of cofactors and vitamins,” and “Lipid metabolism,” and pathways related to sub-health and disease, including “Xenobiotics biodegradation and metabolism,” “Aging,” “Substance dependence,” “Infectious disease: parasitic,” and “Immune disease,” were higher in captive PHs and AWAs than in wild populations. Wild equids, however, had a higher abundance of pathways for metabolism and development, including “Glycan biosynthesis and metabolism,” “Nucleotide metabolism,” “Cell growth and death,” “Translation,” and “Transcription,” as well as pathways about handling various adverse factors such as “Replication and repair,” “Immune system,” and “Environmental adaptation,” than captive populations. This analysis revealed that equids in different environments exhibited apparent divergence in microbiome functions in addition to the diversity and composition of the gut microbiota.

## Discussion

In addition to host phylogeny, environmental factors such as diet, habitat, and lifestyle play a significant role in establishing gut microflora in wildlife (Trevelline et al., 2019; Yu et al., 2022). Meanwhile, the considerable environmental changes that captivity imposes on wildlife remarkably impact their symbiotic gut microbiota (Chong et al., 2019). In this study, we compared the diversity, composition, and function of the gut microbiota of two Equidae species (the Przewalski’s Horse; PH, and the Asian wild ass; AWA) living in different environmental conditions (wild or captive) by 16S rRNA gene sequencing, to explore how the same living environment shapes the gut microflora of different species of Equidae and to reveal the implications of changes in survival environments on equine health from the perspective of the symbiotic microbiome.

The Alpha and Beta diversity of the microflora of PHs and AWAs in the same environment exhibited remarkable similarity, indicating that the cohabitation had a convergence effect on the gut microbiota diversity in different species of Equidae. Equids in captivity had significantly lower Alpha diversity, which is typical with most captive wildlife research — captive animals’ symbiotic microbiome diversity has a considerably decreased tendency than their wild counterparts (Kohl et al., 2014; Wang et al., 2017; Quiroga-González et al., 2021; San Juan et al., 2021). The decline in diversity might be due to the artificial captivity of wildlife greatly hinders their exposure to microorganisms in the natural environment and increases the risk of exposure to antibiotics and anthelmintics, which have detrimental effects on developing the diversity of the microflora (McKenzie et al., 2017; Kunz et al., 2019). At the same time, the limited food variety in the captive lifestyle may also lead to the loss of Alpha diversity in the gut microbiota (McKenzie et al., 2017; Reese and Dunn, 2018). However, low Alpha diversity of the symbiotic microbiome tends to affect the host’s regular metabolism and reduce the host’s ability to resist adverse environments, thus negatively affecting the health of wildlife (Li et al., 2018; Xing et al., 2019). A previous comparative study of captive and wild Tibetan wild asses showed that captivity reduced the Alpha diversity of their gut microbiota and increased their risk of disease (Gao et al., 2019).

The dramatic differences in the bacterial Phylum level between wild and captive equids suggested that various environments had distinctive shaping effects on the host symbiotic microbiota. In the meantime, the captive PHs and AWAs had a considerably higher ratio of *Firmicutes*/*Bacteroidota* (F/B) in their gut microbiota and apparent enrichment of *Actinobacteriota* compared to wild populations. However, many studies have found that as a critical “obesity marker,” a high level of F/B ratio is frequently associated with obesity in humans or animals (Ley et al., 2005; Stojanov et al., 2020). Meanwhile, the relative abundance of *Actinobacteriota* in the intestinal tract is also positively correlated with obesity (Turnbaugh et al., 2009). With the constant supply of “free” food and the severe drop in exercise, obesity has become a prevalent and intractable problem in captive wildlife, which considerably impacts animals’ regular metabolism and health (D’Eath et al., 2009). Therefore, a larger ratio of F/B and a higher relative abundance of *Actinobacteriota* may indicate a greater risk of obesity in captive equids, as well as a threat to the health and conservation of captive threatened equids. It suggests that the F/B ratio of gut microbiota can be utilized as a selective indicator for identifying candidate individuals in the reintroduction of endangered equids and that gut microflora monitoring is required as part of the preliminary preparation for the wildlife reintroduction.

The result of LEfSe analysis of the gut microbial taxa of PHs and AWAs in the two environments showed that the composition and relative abundance of the gut microbiota of distinct species of sympatric equids were essentially the same; however, the gut microflora has dramatically changed while living in different environments, even in the same species. Meanwhile, it is noteworthy that most taxa with significant differences in relative abundance under different environmental conditions play essential roles in host health and disease. Some probiotics that play a critical role in the degradation and fermentation of plant materials were more abundant in wild populations. *Lachnospiraceae* and *Rikenellaceae*, for example, can transform polysaccharides such as plant fibers that animals cannot digest into short-chain fatty acids (SCFAs) that can be absorbed and utilized by the host, mainly acetic, propionic, and butyric acids, which serve as the host’s principal energy source (Boutard et al., 2014; Graf, 2014; Koh et al., 2016). Meanwhile, wild equids showed a higher relative abundance of *Akkermansiaceae* and *Norank_o__WCHB1-41*, and the enterotype represented by these two bacteria can effectively improve the energy and nitrogen utilization efficiency of wildlife (Guo et al., 2021). Due to animals in the field being subject to poorer food conditions, both in quantity and quality, more bacteria associated with energy harvesting and utilization may provide them with a greater capacity to adapt to the harsh wild environment (Yan et al., 2021). In addition, *Christensenellaceae* and *Oscillospiraceae*, more abundant in wild equids, are strongly negatively correlated with metabolic diseases such as inflammation and are essential anti-inflammatory beneficial bacteria (Gophna et al., 2017; Waters and Ley, 2019). Notably, wild equids had substantially more *Eubacterium_coprostanoligenes_group*, which was negatively correlated with anxiety index and positively correlated with anxiety reduction. (Chen et al., 2019).

On the contrary, *Enterococcaceae* is a potential pathogenic pathogen, of which the genus *Enterococcus* is a biomarker for psychological diseases such as depression (Nikolova et al., 2021). However, the relative abundance of *Enterococcus* showed a significant increasing trend in captive PHs (Figure 5C). Previous research has shown that captivity implies more confined living spaces, more anthropogenic stimuli, and less social interaction, thus predisposing wildlife to psychological disorders such as depression and anxiety (Ferdowsian et al., 2011; Lecorps et al., 2021). Thus, variations in the relative abundance of relevant bacterial taxa suggest that the captive environment may impair the mental health of threatened equids to some extent. *Planococcaceae* were significantly increased and had the highest relative abundance in captive equids, and an animal model study suggests that elevated levels of this bacterium may be associated with a high-calorie diet and may exacerbate local inflammatory responses (Bai et al., 2020). Forage for captive Equidae is mainly alfalfa hay, which, as a high-quality forage grass worldwide used, has a high carbohydrate and protein content (Scholtz et al., 2009; Bouton, 2012), as well as a constant supply of feed, which may cause a large caloric intake of captive Equidae, resulting in a dramatic increase in the level of *Planococcaceae*. Potentially pathogenic bacteria such as *Micrococcaceae* (Popovici et al., 1978), *Nocardiaceae* (Goodfellow, 2014), *Carnobacteriaceae* (Aranaz et al., 2021), and *Dietziaceae* (Koerner et al., 2009) were more abundant in PHs, AWAs, or both in the captive environment than in wild populations. These results indicated that PHs and AWAs in the wild possessed more “healthy” gut microbiota, whereas captive populations trended more pro-inflammatory and pathogenic bacteria, as well as bacteria associated with psychological disorders.

Microbial functional analysis revealed that equids in captivity exhibited significant enrichment in nutrient metabolisms such as amino acid, lipid, cofactors, and vitamins metabolism, implying again that the food of captive equids is of higher nutritional value and more abundant food resources. Gut microbes play an essential role in addiction formation (Qin et al., 2021). The increase in the “substance dependence” pathway in captive populations may be related to gut microbiota disturbance caused by excess antibiotics and anthelmintics interventions. Meanwhile, the “Xenobiotics biodegradation and metabolism” pathway displayed an unusually high trend in captive populations, which further proved the exposure risk of captive wildlife to exogenous substances such as drugs, thus increasing the probability of gut microbial dysbiosis and even disease. Higher “Infectious disease: Parasitic” and “Immune disease” pathways in captive equids demonstrated significant subhealth status. Captive populations enriched in the “Aging” pathway, indicating that PHs and AWAs in captivity may have shorter lifespans than wild populations, which may be due to more significant captive stress than captive welfare (Mason, 2010). Equids in the wild presented enrichment in pathways such as “Glycan biosynthesis and metabolism,” “Nucleotide metabolism,” and “Cell growth and death,” indicating higher levels of growth and development (Viinikangas et al., 2021), which might be attributed to the higher levels of short-chain fatty acid (SCFA) generating bacteria which are involved in energy-yielding. In addition, wild equids were more abundant in “Replication and repair,” “Environmental adaptation,” and “Immune system” pathways suggesting that wild populations may have more outstanding capabilities to adapt to complex environments and resist pathogens.

## Conclusion

Overall, PHs and AWAs had significantly different microbial diversities, compositions, and predicted functions in the wild and captivity, and all three aspects exhibited considerable convergence in sympatric equids. It suggests that the same living environment could have a convergence effect on the shaping process of the gut microbiota of different species of Equidae to some extent. Furthermore, the gut microflora of wild equids contained more beneficial bacteria associated with energy generation and anti-inflammatory activity, indicating that the evolutionary adaptation of gut microbiota endowed the host with greater adaptability and a healthier physique. However, captive equids showed significantly “unhealthy” microbiota, with lower Alpha diversity, higher levels of pro-inflammatory bacteria, and potentially pathogenic bacteria, including more biomarkers that may represent psychological disorders such as anxiety or depression, as well as enrichment of microbial functions related to exposure to exogenous substances and susceptibility. These results indicate that the captive environment has apparent adverse effects on threatened equids’ physiological and mental health, which may be due to high-intensity drug exposure, artificial stimulation, limited living space, etc. It hints that in the captive breeding of endangered equids, we should pay attention to the suitable combination of coarse and refined grains, avoid excessive human exposure, appropriate use of antibiotics and anthelmintics, and provide ample active space. Our findings demonstrate that environmental factors play a dominant role in establishing the host’s gut microbiota and warrant further consideration and improvement in the future conservation of captive wildlife.

## Data Availability Statement

The datasets presented in this study can be found in online repositories. The names of the repository/repositories and accession number(s) can be found in the article/ Supplementary Material.

## Ethics Statement

The animal study was reviewed and approved by the Ethics and Animal Welfare Committee of Beijing Forestry University (EAWC_BJFU_2021012).

## Author Contributions

DZ, ZZ, DH, and LY conceived and designed the experiments. HJ, YX, and CS performed the sample collection and DNA extraction. ZZ, LT, HW, and LH analyzed the data. ZZ, LT, QZ, JS, and DZ wrote and revised the manuscript. All authors contributed to the article and approved the submitted version.

## Funding

This study was funded by the Beijing Forestry University Outstanding Young Talent Cultivation Project (no. 2019JQ0318).

## Conflict of Interest

The authors declare that the research was conducted in the absence of any commercial or financial relationships that could be construed as a potential conflict of interest.

## Publisher’s Note

All claims expressed in this article are solely those of the authors and do not necessarily represent those of their affiliated organizations, or those of the publisher, the editors and the reviewers. Any product that may be evaluated in this article, or claim that may be made by its manufacturer, is not guaranteed or endorsed by the publisher.

## Footnotes

^1^ http://huttenhower.sph.harvard.edu/lefse/

## Supplementary Material

The Supplementary Material for this article can be found online at: https://www.frontiersin.org/articles/10.3389/fmicb. 2022.832410/full#supplementary-material
